# Quantifying the Value of Perfect Information in Emergency Vaccination Campaigns

**DOI:** 10.1371/journal.pcbi.1005318

**Published:** 2017-02-16

**Authors:** Naomi V. Bradbury, William J. M. Probert, Katriona Shea, Michael C. Runge, Christopher J. Fonnesbeck, Matt J. Keeling, Matthew J. Ferrari, Michael J. Tildesley

**Affiliations:** 1 School of Veterinary Medicine and Science, University of Nottingham, Leicestershire, United Kingdom; 2 School of Life Sciences, University of Warwick, Coventry, United Kingdom; 3 Mathematics Institute, University of Warwick, Coventry, United Kingdom; 4 Center for Infectious Disease Dynamics, Department of Biology, Eberly College of Science, The Pennsylvania State University, University Park, Pennsylvania, United States of America; 5 Department of Biology and Intercollege Graduate Degree Program in Ecology, 208 Mueller Laboratory, The Pennsylvania State University, University Park, Pennsylvania, United States of America; 6 US Geological Survey, Patuxent Wildlife Research Center, Laurel, Maryland, United States of America; 7 Department of Biostatistics, Vanderbilt University School of Medicine, Nashville, Tennessee, United States of America; University of California, Los Angeles, UNITED STATES

## Abstract

Foot-and-mouth disease outbreaks in non-endemic countries can lead to large economic costs and livestock losses but the use of vaccination has been contentious, partly due to uncertainty about emergency FMD vaccination. Value of information methods can be applied to disease outbreak problems such as FMD in order to investigate the performance improvement from resolving uncertainties. Here we calculate the expected value of resolving uncertainty about vaccine efficacy, time delay to immunity after vaccination and daily vaccination capacity for a hypothetical FMD outbreak in the UK. If it were possible to resolve all uncertainty prior to the introduction of control, we could expect savings of £55 million in outbreak cost, 221,900 livestock culled and 4.3 days of outbreak duration. All vaccination strategies were found to be preferable to a culling only strategy. However, the optimal vaccination radius was found to be highly dependent upon vaccination capacity for all management objectives. We calculate that by resolving the uncertainty surrounding vaccination capacity we would expect to return over 85% of the above savings, regardless of management objective. It may be possible to resolve uncertainty about daily vaccination capacity before an outbreak, and this would enable decision makers to select the optimal control action via careful contingency planning.

## Author Summary

In the UK during 2001 there was an outbreak of foot-and-mouth disease (FMD) which cost the economy an estimated £8 billion and led to the culling of approximately 7 million livestock. The main methods used to control the epidemic were movement bans and culling of infected and high-risk livestock. FMD vaccines were available but not used because of concerns about their effectiveness and how their use would affect the UK’s disease-free status. Using the Warwick FMD model, we ran simulations of FMD outbreaks in the UK including ring vaccination as a method of outbreak control with varying levels of vaccine efficacy, time delay between vaccination and conferral of immunity, and vaccination capacity. We applied value of information analysis to these results and found that the most important factor in determining the optimal vaccination strategy was knowledge of the vaccination capacity. In contrast, vaccine efficacy and delay between vaccination and immunity were relatively unimportant from a decision making perspective. This work could inform contingency planning that would lead to cost savings in the event of a future FMD outbreak and could also be applied to other infectious diseases.

## Introduction

During a new outbreak of an infectious disease, epidemiological models are generally utilised to inform policy decisions. However, such models are normally developed and parameterised using data from previous outbreaks. Whilst these models provide useful information, each novel crisis is likely to unfold in a unique way dependent on the factors particular to that epidemic. In the United Kingdom during 2001 there was a major epidemic of foot-and-mouth disease (FMD), a highly infectious disease of cloven-hoofed animals caused by infection with the virus *Aphthae epizooticae*. The most relevant information about the 2001 FMD outbreak came from analysis of the dynamics of that particular outbreak as it occurred [1–3]. Between outbreaks, research can be focused on minimising future outbreak uncertainty. Value of information (VOI) analysis is a method that allows a decision maker to place a value on reducing the level of uncertainty, by measuring how much the expected outcomes from the decision could be improved if uncertainty could be reduced [4]. This allows for the identification of uncertainties that are important to management. Research to resolve those important uncertainties can then be prioritised. Value of information methods were initially developed in economic and process control settings in the 1960s, but have since been applied in health risk management [4], natural resource management [5] and other fields. The application of value of information methods in infectious disease management has only recently been explored [6–8].

The importation of FMD into a previously disease-free nation has the potential to incur large economic losses owing to the export bans of products from FMD-susceptible animals. Therefore, control measures aim to balance achieving disease-free status as rapidly as possible (which can be reinstated no sooner than 3 months after culling of the last infected animal) with minimising livestock losses through culling [9]. The methods used to tackle the UK 2001 FMD outbreak included culling of all livestock on infected premises (IPs) (those with confirmed cases of FMD) as well as those farms thought to be at high risk of being infected, classified as dangerous contacts (DCs). Proximity culling was also implemented, including culling of livestock on contiguous premises (CPs) (those sharing a border with an IP) and ring culling in certain parts of the country [10]. Around 7 million animals were slaughtered to try to prevent the spread of infection. The agricultural industry and related rural and tourism industries were affected with an estimated total cost to the UK economy of £8 billion [10].

Routine FMD vaccination is not permitted under EU legislation. Emergency vaccination may be used during an outbreak to control the spread of disease or to protect certain livestock but its use is contentious. Also, any previously FMD-free country introducing vaccination during an outbreak will be subject to a change in their OIE (World Organisation for Animal Health) FMD-free status and this can have serious repercussions for their export markets [11]. Owing to this, and to the fact that the limited resources available at the time were thought to be insufficient to have a significant effect, use of emergency vaccination was discussed during the 2001 epidemic but never implemented. Since 2001, modelling work on the FMD outbreak has estimated that, had ring vaccination been implemented alongside culling of IPs and DCs, there would have been a decrease in the duration of the outbreak and the number of farms infected [12–14].

The choice of whether to implement FMD vaccination or not is hampered by uncertainty [10]. There will always be unresolvable uncertainty due to the stochasticity that results in differences between outbreaks of the same disease. However, epistemic uncertainty [15], which encompasses scientific uncertainty about the structure of a model due to incomplete knowledge, can be reduced through research [16]. Resolving the important uncertainties leads to higher expected achievement of management objectives. Short-term learning, via adaptive management (AM), may reduce epistemic uncertainty and lead to long-term improvements in management [5]. Previous work on FMD suggests that a temporally-static approach to management would result in severe strategies, such as culling of IPs, DCs and CPs, being optimal in high density farming regions [6]. However, an approach that uses adaptive management may allow for less severe culling strategies to be introduced initially, under certain conditions. Once uncertainty regarding disease spread had been resolved, additional culling would only be performed if necessary. Simulations of this adaptive strategy were found to result in a significant overall saving in average outbreak cost [6]. It is therefore crucial to quantify the VOI during the early stages of a disease outbreak in order to inform policy makers regarding how much they should invest in “learning” about how a disease is spreading and the resources available for control so that appropriate interventions can be chosen that will minimise the overall cost of the outbreak.

This research considers the impact of resolving uncertainty surrounding emergency vaccination prior to an FMD epidemic in the UK. We investigated two different types of uncertainty associated with vaccination: 1) uncertainty surrounding emergency vaccine deployment in relation to the number of herds and total head of cattle for which there would be the capacity to vaccinate each day in the midst of an FMD outbreak; and 2) uncertainty concerning the efficacy of FMD vaccine as, despite some work on the effectiveness of vaccination in endemic countries [11,17], there is still significant uncertainty regarding the time delay from vaccination to immunity and the efficacy of the vaccine at the herd level during an FMD outbreak [18]. Additionally, it is important to have a clearly defined management objective, as this may have an effect upon the choice of control strategy whilst also allowing for stakeholders and policymakers to be presented with performance information that is most relevant to them. In this paper, we establish the optimal vaccination strategy in the event of uncertainty regarding vaccine capacity and efficacy, whilst considering three alternative management objectives: minimising outbreak duration, minimising total head of livestock culled and minimising epidemic cost (see methods section for a description of the cost function). We determine the optimal vaccination strategies in the presence of these uncertainties and explore the expected performance improvement of resolving these uncertainties prior to deployment.

## Results

**Fig 1:**
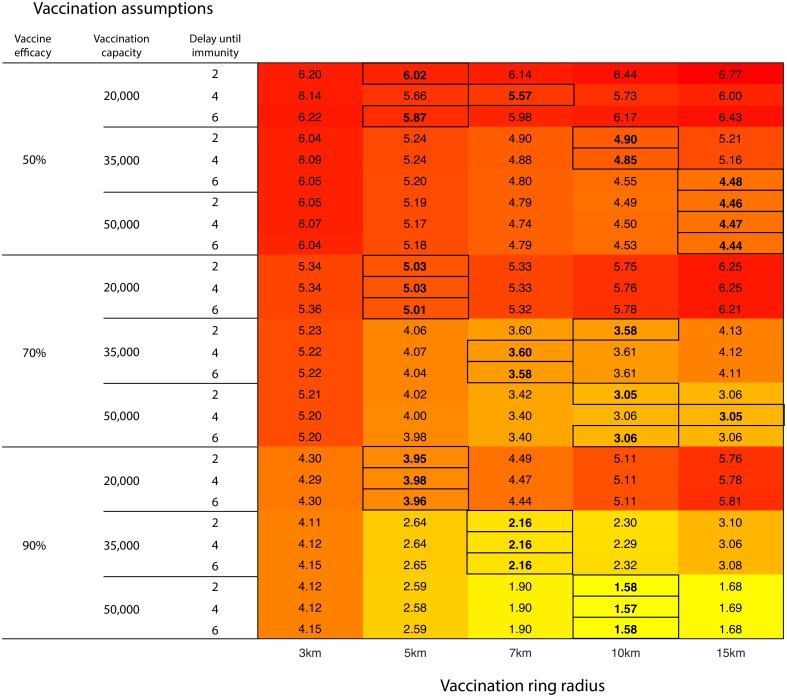
Projected mean livestock culled (million head) under various vaccination ring radii (columns) and vaccination parameterisations (rows). Rows represent different combinations of parameters regarding vaccination efficacy, vaccination capacity (number of animals vaccinated per day), and the delay (in days) from administering vaccination and conferral of immunity. Results have been presented rounded to two decimal places with the optimal strategy selected prior to rounding. Outlined values denote the optimal control action for a given set of vaccination assumptions.

**Fig 2:**
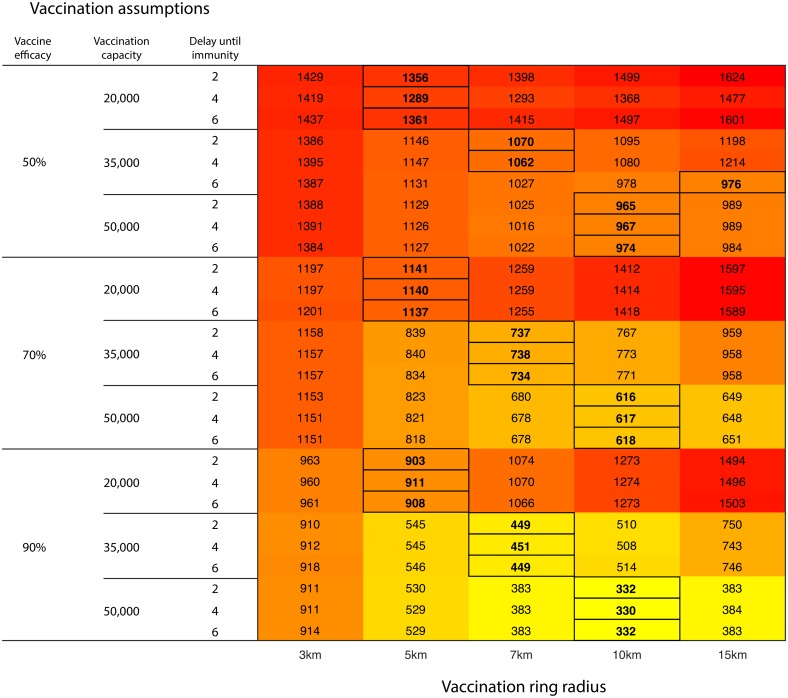
Projected mean outbreak cost (£million) under various vaccination ring radii (columns) and vaccination parameterisations (rows). Rows represent different combination of parameters regarding vaccination efficacy, vaccination capacity (number of animals vaccinated per day), and the delay (in days) from administering vaccination and conferral of immunity. Results have been presented rounded to the nearest integer value with the optimal strategy selected prior to rounding. Outlined values denote the optimal control action for a given set of vaccination assumptions.

Our model results indicated that an IPDC control strategy would result in an average of 7.96 million head of livestock culled (95% prediction interval 5.93–10.26 million head) at a cost of £2.01 billion (95% prediction interval £1.55—£2.52 billion) and a mean outbreak duration of 343 days (95% prediction interval 229–540 days). All vaccination strategies were found to perform better than IPDC culling alone under all combinations of vaccine assumptions and using any of the outcome measures (Figs 1 and 2, S1 Fig).

Vaccination rings of 3km were found to result in the largest number of animals culled and outbreak cost regardless of the vaccination assumptions, unless vaccination capacity was only 20,000 doses per day. In that case, vaccination at 15km resulted in the largest epidemics (Figs 1 and 2). The optimal vaccination strategy was highly dependent upon the daily vaccination capacity for all outcome measures—as the number of doses increased, there was a preference for larger vaccination rings. However, there appears to be little dependence upon either the vaccine efficacy or the time delay to immunity. When daily vaccine capacity was high and vaccine efficacy was low, larger rings were preferred to minimize the number of livestock culled (Fig 1), whilst higher vaccine efficacies generally resulted in smaller rings being optimal for the same outcome measure. Results for the number of livestock culled (Fig 1) and the outbreak cost (Fig 2) follow similar trends because livestock culled was a function of the cost calculation. The converse was true if the outcome measure of interest was minimising outbreak duration, with large rings being optimal when vaccine efficacy and daily capacity were high (S1 Fig). These results highlight the necessity to clearly define the objective of management when determining the control policy that should be implemented [18].

With equal probability weightings for each of the vaccination assumptions (Table 1), the control strategy yielding the worst expected performance in terms of the number of livestock culled and outbreak cost was 3km ring vaccination (5.18 million livestock culled or £1,167 million) and that with the best expected performance was 7km ring vaccination (4.04 million livestock culled or £891 million average cost). Under equal probability weightings the EVPI was 221,900 head or £55 million (5.8% and 6.6% of the expected value in the face of uncertainty respectively). If outbreak duration is the measure of interest, then 10km vaccination is preferred, though the EVPI was only 4.3 days (Table 1).

**Table 1:** Expected livestock culled (million head), outbreak cost (£million) and outbreak duration (days) for each of the control strategies under the assumption of equal weighting across the 27 vaccination parameterisations. Values in blue represent the optimal control strategy to minimise the management objective of interest and values in red represent the worst strategy. The final column shows the EVPI and % EVPI for each cost measure.

*Management objective*	*3km*	*5km*	*7km*	*10km*	*15km*	*EVPI (%)*
Livestock culled	*5*.*18*	4.28	**4.04**	4.09	4.42	0.23 (5.8%)
Cost	*1167*	931	**891**	932	1057	55 (6.6%)
Outbreak duration	*277*.*3*	256.3	247.1	**242.8**	243.9	4.3 (1.8%)

The results indicate that the optimal vaccination strategy is highly dependent upon the daily vaccination capacity. With this in mind, we calculated the EVPXI for all of the model parameters under each management objective (S1–S9 Tables). Resolving uncertainty regarding the time delay to immunity resulted in no benefit in determining the optimal control policy (S3, S6 and S9 Tables), whilst resolving uncertainty in the vaccine efficacy resulted in modest gains (10.8% of the EVPI for outbreak duration (S1 Table) and 7.3% for livestock culled (S4 Table)). However, if one were able to resolve the uncertainty regarding the number of animals that could be vaccinated per day, this can result in significant benefits: 88.7% of the EVPI can be recovered when considering outbreak duration (S2 Table), 89.7% for livestock culled (S5 Table) and 96.6% of the EVPI for epidemic cost (S8 Table). This indicates that, prior to a new outbreak of FMD, it is crucial to determine the capacity for administering vaccination, as this can have a significant influence upon the ability to determine the vaccination radius that should be implemented around all infected farms.

**Fig 3:**
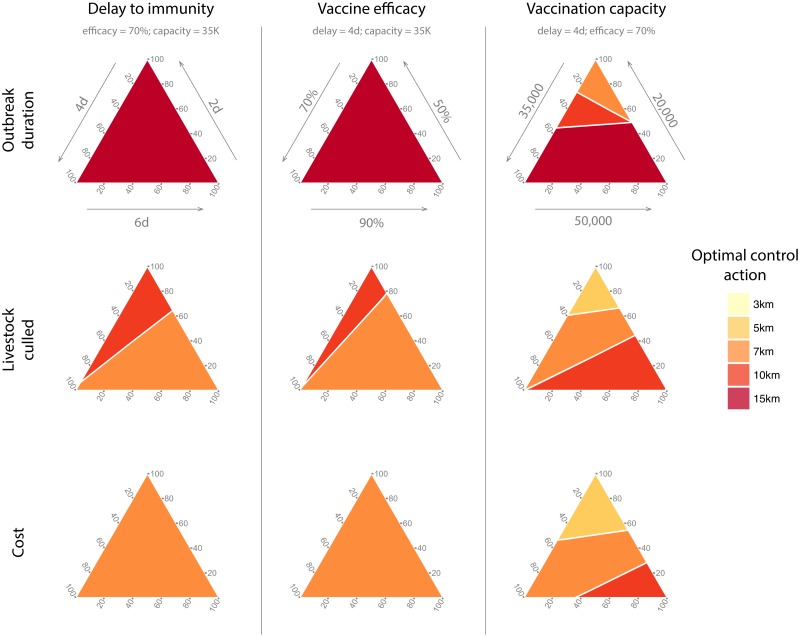
Ternary plots showing the control action which minimises the measure of management success shown in the respective row. Columns represent the specific vaccination assumption that is being varied (with the other two assumptions fixed at intermediate values). The edges of the individual ternary plots are the belief weight axes. Gridlines for tick marks on the belief weight axes run between one axis and the next axis anti-clockwise. Axis labels remain the same down each column. For instance, the ternary plot in the second row and third column shows the control action that minimises the number of livestock culled for different belief weights associated with vaccination capacity (with delay to immunity fixed at 4 days, and vaccine efficacy fixed at 50%). For example, the top vertex of this plot shows that ring vaccination at 5km is optimal when the belief weight for a vaccination capacity of 20,000 doses per day is 100% (and zero on the other two levels).

Finally, we investigated the predictions of the optimal vaccination radius as two of the three vaccination assumptions were fixed at their intermediate values, whilst the weights on the assumptions for the remaining parameter were varied (Fig 3). When vaccine efficacy was set to 70% and capacity was set to 35000 animals per day, 15km and 7km vaccination was optimal to minimise epidemic duration and cost respectively, regardless of the weighting on the three time delay assumptions (Fig 3, left column, top and bottom panels). However, if we were interested in minimising the number of livestock culled, 7km vaccination was optimal unless the weight of belief on a 2 day delay was high, in which case 10km vaccination was optimal (Fig 3, left column, middle panel). A similar result was found when varying the weights on vaccine efficacy, with time delay fixed at 4 days and capacity at 35000 animals per day. Vaccination at 15km and 7km was again optimal for minimising outbreak duration and cost respectively (Fig 3, middle column, top and bottom panels). However, 7km vaccination was optimal for minimising the number of livestock culled, unless the weighting on 50% efficacy was high. In that case, 10km vaccination was again optimal (Fig 3, middle column middle panel). An alternative to fixing two of the three vaccination criteria to their intermediate value is averaging (calculating the mean) across the range of values for these criteria. The results of this analysis are shown in S2 Fig. We find qualitatively similar results in this case, indicating that resolving uncertainty in the number of doses that can be administered per day is key to determining the optimal vaccination strategy.

We saw dramatically different results when we varied the weights on the daily vaccination capacity (fixing time delay to 4 days and efficacy to 70%). In this case, large vaccination radii were found to be optimal when the weighting on the largest capacity, 50000 animals per day, was high, for all management objectives (Fig 3, right column). As the weight of belief on the lowest capacity increased, smaller vaccination radii become optimal. As more doses are available per day to carry out vaccination, it is possible to vaccinate in a larger area around each IP, thus creating a larger zone within which the susceptibility of the population is reduced. As the capacity decreases, the time taken to vaccinate farms in large rings will increase and this will also increase the risk of the virus escaping. This result adds support to the EVPXI results outlined above, that it is crucial to resolve uncertainty regarding vaccination capacity in order to determine the optimal control policy.

## Discussion

In the event of outbreaks of foot-and-mouth disease, vaccination is usually considered as part of a set of control strategies to reduce the impact of the epidemic. However, the adoption of vaccination as an active control measure is limited due to significant uncertainty regarding the effectiveness of vaccination in the field [19] and the resources available to carry out such a vaccination campaign. In the UK 2001 outbreak, the use of vaccination was contentious [10] and ultimately emergency FMD vaccination was not implemented. Despite these uncertainties, vaccination remains part of the UK FMD contingency plan and would be considered for future outbreaks.

In this paper, we have quantified the costs associated with uncertainty regarding three key factors: time delay to immunity after vaccination, the efficacy of the vaccine and the number of animals that can be vaccinated per day. Our results show that if uncertainty could be resolved *a priori*, this would result in an expected decrease of 4.3 days of outbreak duration, 221,900 livestock culled and £55 million based on the 2001 FMD outbreak in the UK. These simulations also show that all simulated vaccination strategies are worth considering in the event of a future outbreak of FMD in the UK as vaccination is expected to reduce the duration of an outbreak, the number of livestock culled and therefore the outbreak cost in comparison to IPDC culling alone. This is in agreement with previous work [12,13].

Using expected value of partial perfect information (EVPXI) analysis we established that there are minimal potential savings to be made through reducing uncertainty in the efficacy of FMD vaccination or the delay between vaccination and conferral of immunity. However, there are larger potential savings to be made by resolving the uncertainty surrounding the daily vaccination capacity within the UK during an FMD epidemic. A clear understanding of daily capacity would also allow policy makers to make more informed decisions regarding the size of the vaccination ring that should be implemented. If there is confidence that vaccination capacity is low (20,000 doses per day), smaller vaccination rings of 5 or 7km are preferential. In contrast, if there is confidence that vaccination capacity is high (50,000 doses per day) then there are the resources to rapidly vaccinate larger areas and 10 or 15km vaccination rings become the better strategy for minimising epidemic impact. By resolving the uncertainty surrounding vaccination capacity, we calculate that the majority of the EVPI (>85%) could be returned regardless of the management objective of interest.

Such a result is relevant to outbreak control, because resolving the biological uncertainty surrounding FMD vaccination is likely to be expensive as it would involve extensive vaccine testing in livestock. Even with such research, there would likely still be unresolved uncertainty as vaccines may vary in efficacy dependent on factors such as the serotype of FMD that has caused the outbreak and the brand of vaccine that is being used. Such a result is also useful prior to an outbreak. It is relatively straightforward to resolve the uncertainty surrounding vaccine capacity within the UK through outbreak planning. Furthermore, our results show that higher capacity is generally better for all objectives. Contingency planning prior to an outbreak allows policy makers an opportunity to prepare and ensure sufficient capacity.

In a real world situation, there may be the resources to vaccinate a known number of livestock daily but this may not be achievable depending on the spatial deployment of vaccination teams in comparison to the dynamics of the outbreak. For example, there may be a large difference between planned capacity and realised capacity if the outbreak is dispersed rather than localised. The model used to run these simulations did not take this partial controllability issue into account. Alternative FMD simulation models such as AusSpread [20] take local resource limitations into account and including this within the Warwick model would give greater confidence in the accuracy of those results on a local level. It would also allow for other resource-limited factors to be considered, such as disposal capacity of culled carcasses.

These simulations focused only on the uncertainty surrounding FMD vaccination as a control strategy whereas, in reality, there are many different uncertainties in an outbreak situation [6]. To conduct an EVPI analysis requires placing a belief weighting on each of these uncertainties, which may not be simple to do in practice. This could be improved if there was more knowledge about the different uncertainties that were considered. For example, knowing more about the range and belief weightings of potential vaccination capacities that could be available in a future FMD epidemic would allow for more accurate outbreak planning.

The use of the cost function in this paper is a simplification of the real economic costs associated with an outbreak. Our main aim in including a cost function was to be able to represent the relative costs of culling compared with vaccination. As compensation costs for culling of livestock (cattle in particular) are generally much higher than costs associated with vaccination, strategies that include significant levels of vaccination may actually be more economical than strategies that involve culling of livestock alone. The vaccination cost estimation was based only on calculations of emergency vaccination cost for herds in the US [21] as there is a dearth of published data in this area. The culling costs were taken from within the compensation range reported from the 2001 outbreak as detailed extensively in the Lessons to be Learned Inquiry [10]. Both were only designed to be representative values and these would need to be updated in a real outbreak scenario. There are also many other economic costs related to an FMD outbreak, such as those arising from export bans and losses to tourism. A more comprehensive cost function could be developed to take into account the wider costs of an FMD epidemic, in particular the economic cost associated with longer export bans as soon as livestock are vaccinated against FMD and the more local impact on businesses in FMD affected areas throughout the duration of the outbreak. However, regardless of the measure used to determine management success, the same conclusion is reached, that resolving uncertainty regarding vaccine capacity is critical in determining the optimal control policy.

In conclusion, these results indicate that emergency vaccination is an important control action to consider during an FMD outbreak situation despite the uncertainty surrounding vaccine behaviour. We show that the level of vaccine efficacy and the time delay to immunity has relatively little importance on the EVPI and the optimal control strategy. Therefore, whilst better information regarding efficacy and time delay will provide more accurate predictions of the number of farms and animals infected, more knowledge in these areas is not vital in order for policy makers and stakeholders to make decisions about the use of vaccination as a control policy. Reliable information on vaccination capacity should be obtained as soon as possible during an outbreak or, better yet, enhanced through contingency planning prior to an outbreak. This approach can also be employed to address similar issues for emergency vaccination campaigns for other diseases.

## Methods

The Warwick FMD model was used to simulate several control measures under a range of scenarios across different levels of uncertainty surrounding vaccination assumptions [1]. This stochastic, fully spatial, premises-based model was developed at the University of Cambridge during the 2001 UK FMD epidemic. Since 2003 it has continued to be developed at the University of Warwick and has been widely used for investigating culling and vaccination strategies during outbreak scenarios [1,13,22–24]. See S1 Appendix for further detail on the Warwick FMD model.

Vaccination does not confer immediate nor complete immunity. In an outbreak scenario, vaccine effectiveness and delay between vaccination and immunity may have an important effect on how useful an emergency vaccination response will be. Equally, during an epidemic, resource limitation may restrict how many doses of vaccination can be delivered daily. The Warwick model was adapted to investigate each of these possibilities.

Previous work suggests that vaccine efficacy during an FMD outbreak can range from 60–85% depending upon the serotype of the virus and the vaccine used [17,25]. In this paper, in order to capture this uncertainty, we considered the possibility that vaccination confers 90%, 70% or 50% immunity. It was assumed that on vaccinated farms the proportion of cattle for which the vaccine was effective became completely immune and the remaining proportion stayed totally susceptible and were capable of infection by, and transmission of, the virus. In other words, for a model with 90% vaccine efficacy, vaccinated farms were assumed to have the same susceptibility and transmissibility as an unvaccinated farm with 10% of the number of cattle.

There is also uncertainty about the time delay between vaccination and the conferral of immunity. Previous work shows that levels of virus neutralising antibodies rise rapidly between 2 and 6 days after vaccination [26]. Therefore, we considered the possibility of a 2, 4 or 6 day delay between vaccination and the conferral of immunity. We assumed that during the delay time the vaccinated animals would be completely susceptible to FMD (and also fully capable of transmission), although this is a somewhat conservative estimate as immunity should build up over this time. After the delay period, the protected cattle were assumed to be completely immune and unable to transmit the virus.

The European Union FMD vaccine bank holds substantial supplies of vaccine, although in the midst of an outbreak it may not be possible for all identified animals to be vaccinated each day owing to resource limitations [27]. As Defra's (Department for Environment Food and Rural Affairs) daily expected capacity of emergency vaccination in the aftermath of the 2001 FMD outbreak was thought to be around 35 000 doses per day [13], we considered the possibility of emergency vaccination capacity of 20 000, 35 000 and 50 000 doses per day.

All combinations of these three parameters (vaccine efficacy, delay and capacity) were considered, giving 27 sets of assumptions regarding vaccination in total. We ran simulations using the FMD model to determine the effectiveness of ring vaccination for a range of ring sizes, in order to determine the optimal vaccination radius that should be introduced in the presence of uncertainty. In the event of ring vaccination being implemented, all farms within a given radius of an IP would be vaccinated, whilst IP and DC culling would also be carried out. Vaccination rings of 3km, 5km, 7km, 10km and 15km were considered. Vaccination in the model takes place firstly in the order in which IPs are reported and then from the outside of each ring moving in towards the centre. Alternative prioritisations for ring vaccination have been investigated elsewhere [13]. For each possible scenario, as well as a control scenario of IP and DC culling only (IPDC), 2000 simulations were conducted using the same state of the outbreak as that on the date that movement restrictions were introduced during the 2001 UK epidemic (23^rd^ February 2001).

In the event of an outbreak of infectious disease, policy makers will make a control decision based upon a set of management objectives. Dependent upon the outbreak scenario, these objectives may range from minimising the duration of the epidemic, the total number of individuals infected or the total economic cost of an outbreak. With this in mind, we have considered three different management objectives, with the caveat that true objectives in the event of an outbreak may be more complex than those investigated here. The first objective we considered was to minimise the duration of the epidemic (in days). This is likely to lead to severe culling strategies being preferred in order to quickly ‘stamp out’ the epidemic but this also causes large livestock losses. Therefore, our second objective of interest was minimising total livestock culled. In this scenario, policy makers may be more likely to favour mass vaccination strategies. However, costs are often an important factor when deciding on control strategies so for the final management objective we considered minimising outbreak cost, focusing on the costs of culling and vaccinating livestock. We used a previously developed cost function designed to measure the cost of culling livestock [5] and included a term for the cost of vaccination. The estimate of the cost of the control measures was calculated using:

$$C=1000{\text{M}}_{culled,cattle}+100{\text{M}}_{culled,sheep}+20{\text{M}}_{vaccinated}$$

Here, *C* is the cost in pounds sterling, *M*_*culled*,*cattle*_ is the total number of cattle culled, *M*_*culled*,*sheep*_ is the total number of sheep culled and *M*_*vaccinated*_ is the total number of cattle vaccinated in the simulation models. From the 2001 Lessons to be Learned Inquiry [10], compensation costs for culled cattle ranged from £150 to £1100, whilst compensation costs for sheep ranged from £32 to £150. In line with previous work [5], we estimated the average compensation costs to be £1000 per culled cow and £100 per culled sheep, which represents an intermediate value in the compensation cost range from 2001. Should vaccination be implemented in the UK, it is likely to be only targeted at cattle owing to the high values associated with cattle herds [13]. We therefore base our vaccination costs on an estimate of the cost of emergency FMD vaccination of small herds of cattle in the United States [21], with an average estimated cost of £20 per vaccinated animal. Whilst we accept that the actual cost associated with livestock epidemics is more complex than that stated here, our aim in this paper is not to determine the actual ‘best’ vaccination policy to implement for a livestock disease outbreak, but to understand the impact of uncertainty in control actions upon a model’s ability to provide policy recommendations.

If all parameter combinations generate model results that are in agreement about the optimal action, then the decision can be made without further analysis. When there is disagreement under varying assumptions, the expected value of perfect information (EVPI) can calculate the theoretical maximum achievable benefit of resolving uncertainty. The EVPI is the difference between the expected value with uncertainty (the action with the best weighted average over all parameter combinations) and the expected value without uncertainty (the weighted average of the optimum outcome over all parameter combinations), and is calculated as:

$$EVPI=E_{s}[{\text{min}}_{a}V(a,s)]-{\text{min}}_{a}E_{s}[V(a,s)]$$

where *a* is the control action taken, *s* is the parameter combination, V_(a,s)_ is the value of action *a* under parameter combination *s*, and min is the minimum over all potential actions for the chosen value function of interest (livestock culled, cost or outbreak duration) [5,6]. The reader should note, especially when comparing to other texts, that here all values are expected to be minimized and hence the EVPI will be a negative value (if the operator was a maximum, EVPI would be a positive value). EVPI analyses were conducted using either outbreak duration, livestock culled or cost as the management objective of interest, and assuming equal belief weightings for each outcome.

The expected value of partial perfect information (EVPXI) can be used to identify how much each individual parameter contributes to the overall decision problem [5]. It is calculated as:

$$EVPXI_{\left(s_{i}\right)}=E_{s_{i}}[{\text{min}}_{a}E_{s_{i}^{c}}[V(a,s_{i},s_{i}^{c})]]-{\text{min}}_{a}E_{s_{i},s_{i}^{c}}[V(a,s_{i},s_{i}^{c})]$$

where *s*_*i*_ is a subset of parameter combinations and s_i_^c^ is its complement [5,6]. We calculated EVPXI for each of the three parameters in turn whilst there remained uncertainty surrounding the other two parameters. This allowed us to identify the expected value of completely resolving uncertainty for that particular parameter.

The optimal control strategy was also calculated across a range of different belief weightings for each of the three management objectives. We considered each parameter independently, by changing that parameter but keeping the other two fixed at the middle value of the three under consideration (e.g. for vaccine efficacy we consider 50%/70%/90% whilst delay remains at 4 days and capacity is fixed at 35,000 doses per day). These middle values were chosen as they were judged to be closest to the true values based on the existing literature [13,17,25,26]. We also calculated the average optimal control strategy by changing one parameter and taking the average results of all the model simulations (e.g. for vaccine efficacy we calculated the mean of all the results for varying time delay to immunity and vaccine capacity as the weight of belief regarding vaccine efficacy varies).

## Supporting Information

S1 Fig Projected mean outbreak duration (days) under various vaccination ring radii (columns) and vaccination parameterisations (rows). Rows represent different combinations of parameters regarding vaccination efficacy, vaccination capacity (number of animals vaccinated per day), and the delay (in days) from administering vaccination and conferral of immunity. Results have been presented rounded to the nearest integer value with the optimal strategy selected prior to rounding. Outlined values denote the optimal control action for a given set of vaccination assumptions.
(TIFF)

S2 Fig Ternary plots showing the control action that minimises the average (mean) measure of management success shown in the respective row. Columns represent the specific vaccination assumption that is being varied. The edges of the individual ternary plots are the belief weight axes. Gridlines for tick marks on the belief weight axes run between one axis and the next axis anti-clockwise. Axis labels remain the same down each column. For instance, the ternary plot in the second row and third column shows the control action that minimises the number of livestock culled for different belief weights associated with vaccination capacity.
(PDF)

S1 Table Values for calculating the expected value of partial perfect information (EVPXI) for the case of minimising outbreak duration when there is uncertainty about vaccine efficacy. Expected value of partial perfect information calculations regarding vaccine efficacy. Values in blue represent the optimal control strategy to minimise the outbreak duration (in days) and values in red represent the worst performing strategy.
(DOCX)

S2 Table Values for calculating the expected value of partial perfect information (EVPXI) for the case of minimising outbreak duration when there is uncertainty about vaccine capacity. Expected value of partial perfect information calculations regarding daily vaccination capacity. Values in blue represent the optimal control strategy to minimise the outbreak duration (in days) and values in red represent the worst performing strategy.
(DOCX)

S3 Table Values for calculating the expected value of partial perfect information (EVPXI) for the case of minimising outbreak duration when there is uncertainty about vaccine delay. Expected value of partial perfect information calculations regarding delay between vaccination and conferral of immunity. Values in blue represent the optimal control strategy to minimise the outbreak duration (in days) and values in red represent the worst performing strategy.
(DOCX)

S4 Table Values for calculating the expected value of partial perfect information (EVPXI) for the case of minimising livestock culled when there is uncertainty about vaccine efficacy. Expected value of partial perfect information calculations regarding vaccine efficacy. Values in blue represent the optimal control strategy to minimise the livestock culled (million head) and values in red represent the worst performing strategy.
(DOCX)

S5 Table Values for calculating the expected value of partial perfect information (EVPXI) for the case of minimising livestock culled when there is uncertainty about vaccine capacity. Expected value of partial perfect information calculations regarding daily vaccination capacity. Values in blue represent the optimal control strategy to minimise the livestock culled (million head) and values in red represent the worst performing strategy.
(DOCX)

S6 Table Values for calculating the expected value of partial perfect information (EVPXI) for the case of minimising livestock culled when there is uncertainty about vaccine delay. Expected value of partial perfect information calculations regarding delay between vaccination and conferral of immunity. Values in blue represent the optimal control strategy to minimise the livestock culled (million head) and values in red represent the worst performing strategy.
(DOCX)

S7 Table Values for calculating the expected value of partial perfect information (EVPXI) for the case of minimising outbreak cost when there is uncertainty about vaccine efficacy. Expected value of partial perfect information calculations regarding vaccine efficacy. Values in blue represent the optimal control strategy to minimise the cost (£ million) and values in red represent the worst performing strategy.
(DOCX)

S8 Table Values for calculating the expected value of partial perfect information (EVPXI) for the case of minimising outbreak cost when there is uncertainty about vaccine capacity. Expected value of partial perfect information calculations regarding daily vaccination capacity. Values in blue represent the optimal control strategy to minimise the cost (£ million) and values in red represent the worst performing strategy.
(DOCX)

S9 Table Values for calculating the expected value of partial perfect information (EVPXI) for the case of minimising outbreak cost when there is uncertainty about vaccine delay. Expected value of partial perfect information calculations regarding delay between vaccination and conferral of immunity. Values in blue represent the optimal control strategy to minimise the cost (£ million) and values in red represent the worst performing strategy.
(DOCX)

S1 Appendix Further detail on the Warwick FMD model. (DOCX)

S1 Data Data analysed and presented in this paper generated from Warwick FMD model simulations. (ZIP)
