# Supplementary figures and images for: Quantifying the Value of Perfect Information in Emergency Vaccination Campaigns
Source: PLoS Comput Biol. 2017 Feb 16;13(2):e1005318. doi: 10.1371/journal.pcbi.1005318 (PMC5312803; doi:10.1371/journal.pcbi.1005318)

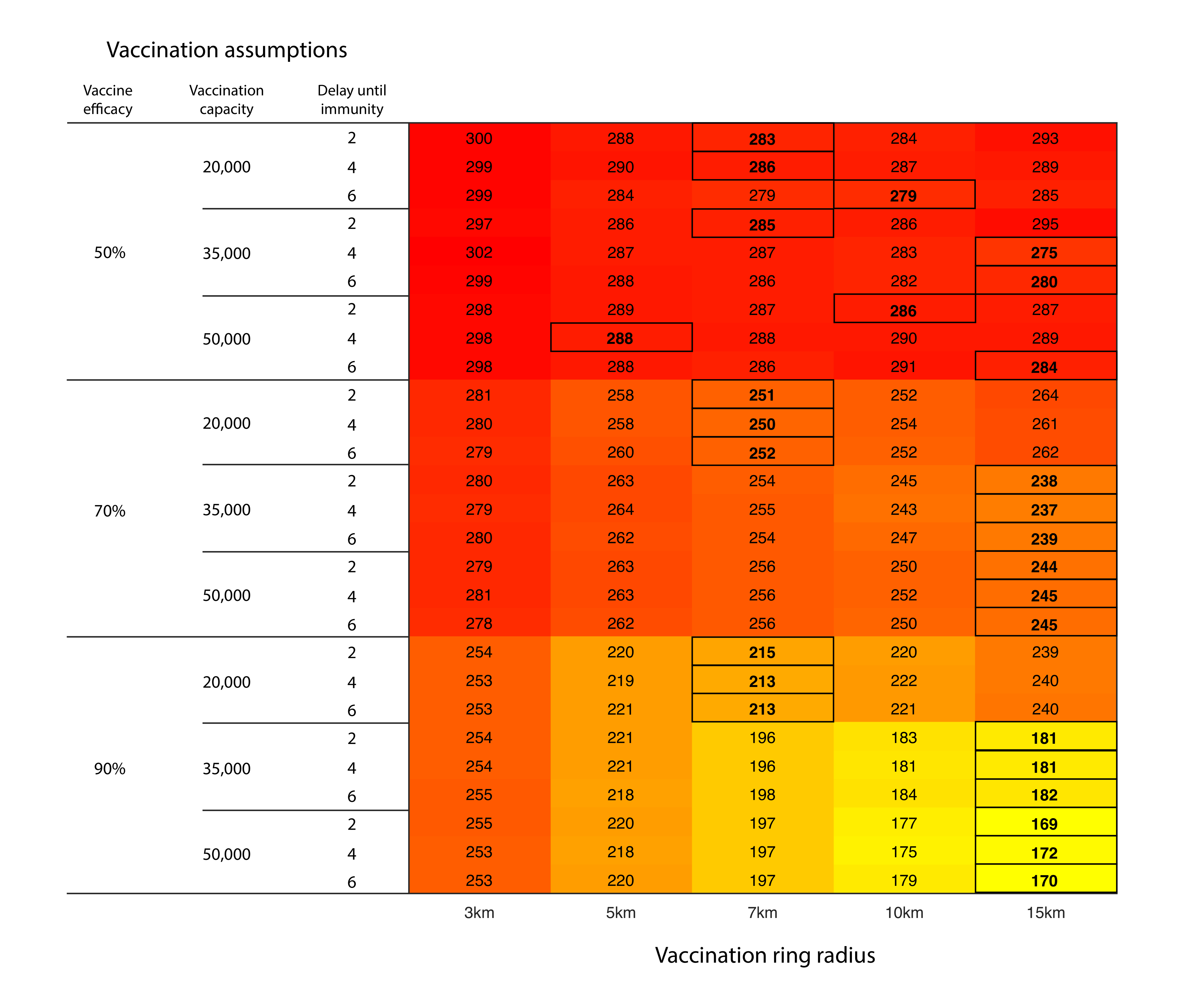

Supplement: S1 Fig — Rows represent different combinations of parameters regarding vaccination efficacy, vaccination capacity (number of animals vaccinated per day), and the delay (in days) from administering vaccination and conferral of immunity. Results have been presented rounded to the nearest integer value with the optimal strategy selected prior to rounding. Outlined values denote the optimal control action for a given set of vaccination assumptions. (TIFF) [file pcbi.1005318.s001.tiff]
