# Supplementary material for: Quantifying the Value of Perfect Information in Emergency Vaccination Campaigns
Source: PLoS Comput Biol. 2017 Feb 16;13(2):e1005318. doi: 10.1371/journal.pcbi.1005318 (PMC5312803; doi:10.1371/journal.pcbi.1005318)
Supplement: S2 Fig — Columns represent the specific vaccination assumption that is being varied. The edges of the individual ternary plots are the belief weight axes. Gridlines for tick marks on the belief weight axes run between one axis and the next axis anti-clockwise. Axis labels remain the same down each column. For instance, the ternary plot in the second row and third column shows the control action that minimises the number of livestock culled for different belief weights associated with vaccination capacity. (PDF) [file pcbi.1005318.s002.pdf]

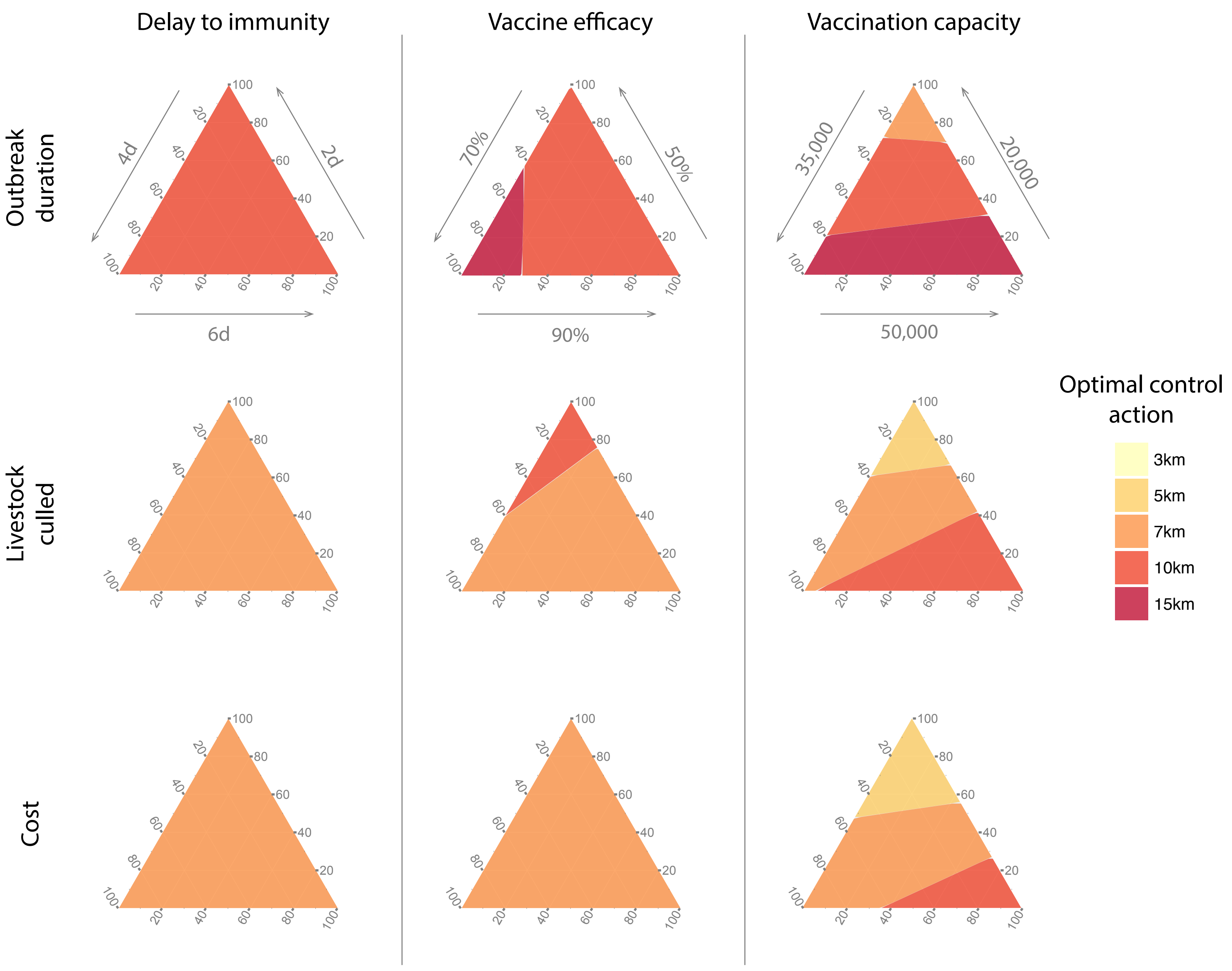

Delay to immunity
Vaccine efficacy
Vaccination capacity
Outbreak duration
Livestock culled
Cost
4d
2d
6d
70%
50%
90%
35,000
20,000
50,000
Optimal control action
3km
5km
7km
10km
15km
