# Supplementary material for: Quantifying the Value of Perfect Information in Emergency Vaccination Campaigns
Source: PLoS Comput Biol. 2017 Feb 16;13(2):e1005318. doi: 10.1371/journal.pcbi.1005318 (PMC5312803; doi:10.1371/journal.pcbi.1005318)
Supplement: S2 Table — Expected value of partial perfect information calculations regarding daily vaccination capacity. Values in blue represent the optimal control strategy to minimise the outbreak duration (in days) and values in red represent the worst performing strategy. (DOCX) [file pcbi.1005318.s004.docx]

| Probability weighting | Efficacy | Doses | | Delay | 3km | 5km | 7km | 10km | 15km |  | Best |
| --- | --- | --- | --- | --- | --- | --- | --- | --- | --- | --- | --- |
| 0.33 | 50% | **20000** | | 4 | *277.4* | 255.5 | **249.1** | 252.3 | 263.6 |  | 249.1 |
| 0.33 | 50% | **35000** | | 4 | *277.6* | 256.6 | 245.8 | 237.2 | **234.3** |  | 234.3 |
| 0.33 | 50% | **50000** | | 4 | *276.9* | 256.8 | 246.6 | 239.0 | **233.8** |  | 233.8 |
|  |  |  | |  |  |  |  |  |  |  |  |
| Weighted average |  |  | |  | *277.3* | 256.3 | 277.1 | **242.8** | 243.9 |  | 293.0 |
|  | | | | | | | | | | | |
| EVPXI | | | 3.8 | | | | | | | | |
| Percentage of total EVPI | | | 88.7% | | | | | | | | |
