# Supplementary material for: Quantifying the Value of Perfect Information in Emergency Vaccination Campaigns
Source: PLoS Comput Biol. 2017 Feb 16;13(2):e1005318. doi: 10.1371/journal.pcbi.1005318 (PMC5312803; doi:10.1371/journal.pcbi.1005318)
Supplement: S7 Table — Expected value of partial perfect information calculations regarding vaccine efficacy. Values in blue represent the optimal control strategy to minimise the cost (£ million) and values in red represent the worst performing strategy. (DOCX) [file pcbi.1005318.s009.docx]

| Probability weighting | Efficacy | Doses | | Delay | 3km | 5km | 7km | 10km | 15km |  | Best |
| --- | --- | --- | --- | --- | --- | --- | --- | --- | --- | --- | --- |
| 0.33 | **50%** | 35,000 | | 4 | *1401.7* | 1201.3 | **1147.4** | 1158.1 | 1227.8 |  | 1147.4 |
| 0.33 | **70%** | 35,000 | | 4 | *1169.0* | 932.5 | **891.1** | 934.0 | 1067.0 |  | 891.1 |
| 0.33 | **90%** | 35,000 | | 4 | *928.9* | 660.6 | **634.0** | 705.2 | 875.8 |  | 634 |
|  |  |  | |  |  |  |  |  |  |  |  |
| Weighted average |  |  | |  | *1166.5* | 931.5 | **890.8** | 932.4 | 1056.9 |  | 890.8 |
|  | | | | | | | | | | | |
| EVPXI | | | 0 | | | | | | | | |
| Percentage of total EVPI | | | 0% | | | | | | | | |
