# Supplementary material for: Quantifying the Value of Perfect Information in Emergency Vaccination Campaigns
Source: PLoS Comput Biol. 2017 Feb 16;13(2):e1005318. doi: 10.1371/journal.pcbi.1005318 (PMC5312803; doi:10.1371/journal.pcbi.1005318)
Supplement: S8 Table — Expected value of partial perfect information calculations regarding daily vaccination capacity. Values in blue represent the optimal control strategy to minimise the cost (£ million) and values in red represent the worst performing strategy. (DOCX) [file pcbi.1005318.s010.docx]

| Probability weighting | Efficacy | Doses | | Delay | 3km | 5km | 7km | 10km | 15km |  | Best |
| --- | --- | --- | --- | --- | --- | --- | --- | --- | --- | --- | --- |
| 0.33 | 50% | **20000** | | 4 | 1196.1 | **1127.3** | 1231.9 | 1380.9 | *1552.8* |  | 1127.3 |
| 0.33 | 50% | **35000** | | 4 | *1153.3* | 841.4 | **746.4** | 777.2 | 944.5 |  | 746.4 |
| 0.33 | 50% | **50000** | | 4 | *1150.3* | 825.7 | 694.2 | **639.1** | 673.2 |  | 639.1 |
|  |  |  | |  |  |  |  |  |  |  |  |
| Weighted average |  |  | |  | *1166.5* | 931.5 | **890.8** | 932.4 | 1056.9 |  | 837.6 |
|  | | | | | | | | | | | |
| EVPXI | | | 53.2 | | | | | | | | |
| Percentage of total EVPI | | | 96.6% | | | | | | | | |
