# Supplementary material for: Quantifying the Value of Perfect Information in Emergency Vaccination Campaigns
Source: PLoS Comput Biol. 2017 Feb 16;13(2):e1005318. doi: 10.1371/journal.pcbi.1005318 (PMC5312803; doi:10.1371/journal.pcbi.1005318)
Supplement: S9 Table — Expected value of partial perfect information calculations regarding delay between vaccination and conferral of immunity. Values in blue represent the optimal control strategy to minimise the cost (£ million) and values in red represent the worst performing strategy. (DOCX) [file pcbi.1005318.s011.docx]

| Probability weighting | Efficacy | Doses | | Delay | 3km | 5km | 7km | 10km | 15km |  | Best |
| --- | --- | --- | --- | --- | --- | --- | --- | --- | --- | --- | --- |
| 0.33 | 50% | 35,000 | | **2** | *1166.1* | 934.6 | **897.3** | 941.0 | 1071.3 |  | 897.3 |
| 0.33 | 50% | 35,000 | | **4** | *1165.8* | 927.5 | **883.1** | 925.7 | 1056.0 |  | 883.1 |
| 0.33 | 50% | 35,000 | | **6** | *1167.7* | 932.2 | **892.1** | 930.6 | 1043.3 |  | 892.1 |
|  |  |  | |  |  |  |  |  |  |  |  |
| Weighted average |  |  | |  | *1166.5* | 931.5 | **890.8** | 932.4 | 1056.9 |  | 890.8 |
|  | | | | | | | | | | | |
| EVPXI | | | 0 | | | | | | | | |
| Percentage of total EVPI | | | 0% | | | | | | | | |
