# Supplementary material for: Quantifying the Value of Perfect Information in Emergency Vaccination Campaigns
Source: PLoS Comput Biol. 2017 Feb 16;13(2):e1005318. doi: 10.1371/journal.pcbi.1005318 (PMC5312803; doi:10.1371/journal.pcbi.1005318)
Supplement: S1 Appendix — (DOCX) [file pcbi.1005318.s012.docx]

**S1 Appendix**

The basis of the Warwick FMD model is that premises can be in one of five different epidemiological states: 1) susceptible, 2) infected but not yet infectious, 3) infectious, 4) reported infectious whereupon the livestock on the farm are targeted for culling, and 5) culled. The daily probability of infection, *P_i_*, of a susceptible premises, *i*, is calculated using:

$$P_{i}=1-\text{exp}\left(-S_{i}\sum_{j\in I} T_{j}K\left(d_{ij}\right)\right)$$

The probability of infection on any particular premises depends upon the susceptibility (*S_i_*) of that premises and the transmissibility (*T_j_*) of the set of ***I*** surrounding infectious premises. The between-farm transmission kernel, *K(d_ij_)* scales the probability of transmission dependent on the distance between farms, *d_ij_*.

The susceptibility and transmissibility of premises depends on both the size of the herd and the composition of the livestock present in each farm, as the epidemiology of FMD infection differs between cattle and sheep. For each premises, *S_i_* and *T_j_* are computed as follows:

$$S_{i}=s_{cattle}N_{cattle,i}^{p_{c}}+s_{sheep}N_{sheep,i}^{p_{s}}$$

$$T_{j}=t_{cattle}N_{cattle,j}^{q_{c}}+t_{sheep}N_{sheep,j}^{q_{s}}$$

 where the parameters *s* and *t* represent the per-head susceptibility and transmissibility of the respective livestock, *N_cattle,i_* and *N_sheep,i_* correspond to the number of cattle and sheep on farm, *i*, and the power law parameters, *p_s_*, *p_c_*, *q_s_* and *q_c_*, allow for the modelling of a non-linear relationship between herd size and the susceptibility and transmissibility of a premises. Pigs were the infected species on only 18 farms in the UK 2001 epidemic and therefore we have little information as to the susceptibility or transmissibility of pigs for this strain of FMD, and are therefore here. However, the effect of an FMD epidemic in the UK pig population has been investigated in detail in previous work [1] .

As with previous uses of this model [2–4] it has been assumed that premises that have been infected have a latent period of 5 days before they become infectious. Once a farm becomes infectious there will be a further delay of 4 days before symptoms are noticed and the farm is reported as infected. For the purposes of this paper, we assume an efficient 24/48 hour policy for culling, whereby all IPs are culled within 24 hours of being reported and all associated DCs are culled within 48 hours. Once a farm has been depopulated, it is disinfected and it is assumed that no further ongoing transmission can occur. Within these simulations only the culling of IPs and DCs is considered, and only cattle are assumed to receive vaccination. The impact of these assumptions upon epidemiological predictions has been investigated extensively elsewhere (1,4).

**References**

1. Tildesley MJ, Deardon R, Savill NJ, Bessell PR, Brooks SP, Woolhouse ME., et al. Accuracy of models for the 2001 foot-and-mouth epidemic. Proc R Soc Lond B Biol Sci. 2008 Jun 22;275(1641):1459–68.

2. Keeling MJ, Woolhouse MEJ, Shaw DJ, Matthews L, Chase-Topping M, Haydon DT, et al. Dynamics of the 2001 UK Foot and Mouth Epidemic: Stochastic Dispersal in a Heterogeneous Landscape. Science. 2001 Oct 26;294(5543):813–7.

3. Keeling MJ, Woolhouse MEJ, May RM, Davies G, Grenfell BT. Modelling vaccination strategies against foot-and-mouth disease. Nature. 2003 Jan 9;421(6919):136–42.

4. Tildesley MJ, Savill NJ, Shaw DJ, Deardon R, Brooks SP, Woolhouse ME, et al. Optimal reactive vaccination strategies for a foot-and-mouth outbreak in the UK. Nature. 2006;440(7080):83–6.
